# Contamination of Honeybee (*Apis mellifera* L.) Royal Jelly by Pesticides and Sample Preparation Methods for Its Determination: A Critical Appraisal

**DOI:** 10.3390/foods12193612

**Published:** 2023-09-28

**Authors:** Adrián Fuente-Ballesteros, Patricia Brugnerotto, Vinh Dinh Nguyen, Ana C. O. Costa, José Bernal, Ana M. Ares

**Affiliations:** 1 Analytical Chemistry Group (TESEA), I.U. CINQUIMA, Faculty of Sciences, University of Valladolid, 47011 Valladolid, Spain; adrian.fuente.ballesteros@uva.es (A.F.-B.); jose.bernal@uva.es (J.B.); 2 Laboratory of Food Chemistry, Department of Food Science and Technology, Federal University of Santa Catarina, Florianópolis 88034-001, SC, Brazil; patriciabrugnerotto@gmail.com (P.B.); ana.costa@ufsc.br (A.C.O.C.); 3 Faculty of Chemistry, TNU-University of Sciences, Tan Thinh Ward, Thai Nguyen City 25000, Vietnam; vinhnd@tnus.edu.vn

**Keywords:** QuEChERS, solid-phase extraction, insecticide, acaricide, bee product, sample treatment

## Abstract

Pesticides can easily enter the food chain, harming bee populations and ecosystems. Exposure of beehive products to various contaminants has been identified as one of the factors contributing to the decline in bee populations, and multiple food alerts have been reported. Despite this fact, royal jelly, a valuable bee product with nutritional and functional properties, has received less attention in this context. Pesticide residues of different chemical class can contaminate royal jelly when foraging bees collect pollen or nectar from pesticide-treated flowers, or in some cases, due to its frequent and inappropriate use in the treatment of mites in beehives. To monitor this issue and also make it more reliable, it is crucial to develop effective sample preparation methods for extracting pesticides from royal jelly for subsequent analysis. In this context, this review provides information about sample preparation methods (solid-phase extraction, solvent extraction, and QuEChERS—quick, easy, cheap, effective, rugged and safe) and analytical methods that have been validated or improved to extract and analyze pesticides, respectively, in royal jelly samples of different origins. Finally, future perspectives are discussed. With this background, we aim to provide data that can guide future research related to this topic.

## 1. Introduction

The consumption of apicultural products such as honey, royal jelly, propolis, and bee pollen is experiencing a surge in popularity. This fact can be attributed to the bioactive compounds found in these products, which have been linked to many health benefits [1,2]. Among these products, royal jelly, a dense and creamy substance secreted by nurse bees from their mandibular glands, has gained significant attention due to its multifaceted biological functions, including its antioxidant, anti-inflammatory, antiviral and antibacterial properties [3,4,5,6,7]. Nevertheless, the potential contamination of bee products, including royal jelly, with pesticides or antibiotics through environmental factors and beekeeping practices, is a growing concern [8]. This contamination not only undermines their perception of healthy products, but also poses a potential risk to consumers [9].

Pesticides used near beehives can disperse in the air and deposit on plants and flowers that bees rely on for nectar and pollen collection [10]. Additionally, pesticide residues can persist in the environment for an extended period, eventually contaminating royal jelly [11,12]. It was demonstrated that 30 out of 176 analyzed pesticides were detectable in different royal jelly samples [13]. The presence of residues in royal jelly depends on the application methods and exposure routes. There are differing viewpoints regarding pesticide residue transfer to royal jelly. Some studies suggest that pesticide residues, such as coumaphos and chlorfenvinphos, may accumulate in beeswax but transfer in trace amounts to royal jelly or do not migrate significantly [14]. This may be due to the filtration process performed by nurse bees during royal jelly secretion, reducing the concentration of toxic substances [15,16] or the rapid metabolism of chemicals [17].

While pesticide residue determination in honey, bee pollen and beeswax has been extensively studied [18], there is a scarcity of publications specifically addressing pesticides in royal jelly [11,13,19,20]. The first article for pesticide analysis in royal jelly was published in the literature in 2001 [21]. Some authors have reported that the available information on royal jelly is insufficient, highlighting the complexity of residue analysis and questioning the reliability of the available analytical methods [22]. Moreover, recently, the problem of royal jelly contamination has been primarily focused on antibiotics (such as chloramphenicol, sulfonamides, fluoroquinolones, tetracyclines, aminoglycosides or macrolides) [23,24,25,26,27,28,29,30], forgetting about other pollutants such as pesticides. It should be noted that residue analysis methods for bee products, including royal jelly, generally employ multi-residue approaches, which begin with sample homogenization using an appropriate solvent. Subsequently, purification and clean-up steps are conducted through solid-phase extraction (SPE), culminating in a final chromatographic determination step. However, the complex nature of bee products poses challenges such as the interference of co-eluted matrix components with residue peaks, inaccurate quantification and the occurrence of false positive peaks. To address the issues, extensive clean-up procedures are necessary to mitigate these adverse effects.

Royal jelly is a complex viscose substance composed of 57–70% water, 9–18% proteins, 3–8% lipids, 6–18% hydrocarbons, 0.8–3.0% minerals and small amounts of vitamins [22,30,31,32,33]. The high protein and water content might cause a notable matrix effect due to matrix interferents. The collection of royal jelly involves stimulating bees to produce this substance and then carefully extracting it from the cells. It is a process that requires experience and knowledge in beekeeping, along with a responsible approach towards the conservation of bees and their environment [30]. Compared to other bee products, the production of royal jelly is relatively low. Worker bees produce royal jelly in limited quantities, exclusively intended for feeding queen bee larvae and a small number of young worker bees; this fact results in a small amount being available for analysis [34]. Additionally, collecting royal jelly requires careful handling to avoid hive damage and disruption of bees’ normal activities, making it a meticulous and labor-extensive task. Royal jelly is stored and processed in special cells affecting the distribution and accumulation of pesticides compared to other bee products that are stored in honeycomb cells. All these factors have significantly increased the price of royal jelly and complicated the process of pesticide extraction and identification. As a result, there is a lack of reliable data on pesticide residues in royal jelly, making it a less extensively studied bee product [15,21].

In this sense, the objective of this study is to develop a critical review of the different families of pesticides that have been usually detected in royal jelly as well as the main sample preparation methods available in the literature to study pesticides in royal jelly, highlighting the large information gap that exists in this area. Readers interested in more specific details, such as the determination techniques mostly employed, and/or royal jelly composition, can refer to some of the mentioned publications and to the related literature.

## 2. Contamination of Royal Jelly with Pesticides

Pesticides are chemical or biological substances used to control or eliminate organisms considered harmful. They are primarily used in agriculture to protect crops, prevent vector-borne diseases, control urban pests, and ensure food quality [15]. The pesticides can be classified based on their chemical origin, persistence, toxicity, chemical structure or target action [10,35,36]. Within the pesticide family, this review has focused on the study of four major families (see Table 1) due to their higher occurrence and direct impact on bee-related matrices [20], specifically on royal jelly [37].

The approval of active substances is based on comprehensive scientific risk assessments. The Food and Agriculture Organization (FAO) Panel of Experts on Pesticide Residues in Food and the Environment evaluates pesticide residue data to establish maximum residue limits (MRLs) for food and feed products [38]. Nevertheless, the absence of fundamental data regarding the transfer of residues from queen cells to royal jelly and the appropriate time intervals between acaricide treatment and royal jelly production poses a barrier to establishing precise limitations [39]. In the European Union (EU), MRLs for food and feed are regulated by the Commission Regulation (EC) No. 396/2005, along with subsequent amendments. However, specific MRLs for apicultural products other than honey have not yet been established or included [40]. The challenge arises from the absence of specific regulations focused on royal jelly, as the existing regulations mainly address honey and other apiculture products in a general manner [15].

A few studies have reported either the absence or negligible levels of pesticide residues in royal jelly [41]. In a study conducted by Böhme et al. [42], a field experiment was designed to simulate real-life conditions, representing a worst-case scenario. Adult honeybees were fed with a pollen-honey diet containing a cocktail of 13 commonly used pesticides (insecticides, herbicides, and fungicides) in high concentrations (34–920 μg/kg). Upon analyzing the royal jelly, authors concluded that the ingestion of multiple pesticides by bees did not impact the development of the queen or young larvae. Interestingly, Martínez-Domínguez et al. (2014) [43] validated a multi-class method for studying 127 pesticides in royal jelly by gas chromatography coupled to triple quadrupole tandem mass spectrometry. The method was applied to the analysis of six royal jelly commercial products (liquid and capsule preparations) and no pesticides were detected above the limits of detection. Valverde et al. (2018) [3] and Zheng et al. (2018) [44] developed and validated methods for determining pesticides in 12 and 10 royal jelly samples, respectively, but no residues were found in the samples. Considering these findings, Milone et al. (2021) [45] hypothesized that royal jelly maintains its pesticide-free status as the nurse bees act as a buffer for chemical residues. However, the effects of pesticides can manifest in the quality of royal jelly produced by nurse bees [45,46] and more interestingly, contamination could occur through migration, as bees can transfer contaminants to each other via trophallaxis within the colony, subsequently transmitting the contaminants to larvae through their food.

### 2.1. Insecticides

Insecticides encompass a wide variety of compounds, including organochlorines, organophosphates, carbamates, and neonicotinoids. Neonicotinoids play a significant role in agriculture due to their potent effectiveness against pests and insects, acting as neurotoxic substances in the insect’s central nervous system [47]. The primary approach commonly employed to control pests involves the application of insecticide-coated seeds, as the majority of pests reside in the soil during the planting phase [48]. However, the utilization of neonicotinoid active substances in the seeds of crops such as cotton, corn, and sunflowers has sparked a hypothesis linking this particular class of pesticides to the occurrence of colony collapse disorder syndrome [10]. Different neonicotinoids have been studied in royal jelly [3,19,34,49], due to the potential presence in crops visited by bees, but in most cases, no neonicotinoids were detected. Seven neonicotinoids (dinotefuran, nitenpyram, thiamethoxam, clothianidin, imidacloprid, acetamiprid and thiacloprid) were investigated in royal jelly-based products from different Spanish regions (*n =* 12), but no residues were detected [3]. This finding does not suggest any limitation on the method’s applicability, as the European Commission established MRLs for these compounds in honey and related matrices, including royal jelly (10–200 μg/kg) [40]. Likewise, Hong et al. (2019) [47] developed a method for the simultaneous determination of ten neonicotinoids (pymetrozine, dinotefuran, nitenpyram, thiamethoxam, flonicamid, imidacloprid, clothianidin, imidaclothiz, acetamiprid and thiacloprid) and two metabolites (4-trifluoromethylnicotinamide and N-desmethylacetamiprid) in royal jelly. They validated the method, obtaining good recoveries (73–107%), and applied it to 60 royal jelly samples, detecting no neonicotinoid insecticides.

Residues of *p*-dichlorobenzene *(p*-DCB), an insecticide used against the *Galleria mellonella* wax moth, have been detected in royal jelly coming from honeycombs [50]. It was found that the levels of *p*-DCB in honey were notably lower than those in royal jelly. In fact, in some cases, royal jelly contained hundreds of times more residues of *p*-DCB compared to honey extracted from the same comb. The maximum concentration of *p*-DCB in royal jelly was 1520 μg/kg [50]. Considering that the usual daily dosage of royal jelly is expected to be lower than 0.5 g, an individual weighing 70 kg would consume approximately 0.014% of the acceptable daily intake. While this amount is considered too insignificant to pose any health concerns for consumers, it is noteworthy that these residues in royal jelly are undesirable due to the absence of established MRLs for hive products [50].

In other studies, a method was developed and validated to determine potential residues of thiamethoxam and clothianidin in royal jelly samples (*n =* 11) available from an online shopping website [34]. Clothianidin was never detected, but thiamethoxam was found in three of them with a concentration comprised between 0.15 and 0.25 μg/kg. Clothianidin is an active substance in insecticidal formulations and also the most toxic metabolite of thiamethoxam, and both neonicotinoids have been demonstrated to have adverse effects on queens [51,52].

### 2.2. Acaricides

To effectively control mite infestations, beekeepers often rely on synthesized substances for crop protection and livestock. Acaricides such as amitraz, cymiazole, bromopropylate, τ-fluvalinate, flumethrin, coumaphos, and malathion have been extensively used by beekeepers worldwide. Various formulations, including Apistan^®^ (containing τ-fluvalinate as the active ingredient), Perizin^®^ (containing coumaphos), Check-Mite^TM^ (containing coumaphos), Bayvarol^®^ (containing flumethrin) and Apiguard^®^ (containing thymol), have gained approval in numerous European countries [53]. Another widely used acaricide in beehives is amitraz, which has attracted interest due to its degradation products, particularly 2,4-dimethylaniline [30]. However, it is important to note that while some substances like amitraz have received approval in specific countries, others like malathion have not been approved at all [10]. Since 1988, τ-fluvalinate has been employed for the control of *Varroa destructor* mites, which represent one of the primary pests affecting honeybees. Typically, it is administered in the form of strips or sheets placed inside beehives, allowing bees to come into contact with it as they move around. The presence of residues from these compounds poses a significant hazard to consumer health, including the potential for mutations or cellular degradation. This issue stems from both direct contamination resulting from beekeeping practices and indirect contamination through environmental sources. The latter option is associated with the presence of τ-fluvalinate in royal jelly, as the widespread use and extensive distribution of pesticides have led bees to consume contaminated flowers, subsequently transferring the contaminants to the royal jelly. In a study conducted by Karazafiris et al. (2022) [39], residues of coumaphos and τ-fluvalinate were found in royal jelly produced from colonies under chemical treatment using artificial plastic queen cells, suggesting that acaricides may be transferred from wax to royal jelly. A parallel investigation [21] performed a similar test by applying pesticides inside the beehives, either in the form of aerosol (coumaphos) or as plywood inserts (τ-fluvalinate). No residues were detected when plywood inserts impregnated with τ-fluvalinate were used, but coumaphos residues were found in the range of 10–92 μg/kg. Lastly, Notardonato et al. (2014, 2016) [54,55] screened five acaricides (τ-fluvalinate, bromopropylate, fipronil, amitraz and coumaphos) in one royal jelly sample finding 81 μg/kg of bromopropylate and in homemade honey foods. However, the results are inconclusive due to the low number of analyzed samples and the lack of information about them.

### 2.3. Herbicides

Under field conditions, bees are often subjected to prolonged exposure to multiple pesticides, which can substantially harm their colonies. Herbicides, formulated to impede the growth, development, or survival of unwanted plants, play a vital role in enabling desired crops or vegetation to flourish unhindered by competition. The herbicides are extensively utilized in certain regions, like Brazil, making them one of the most prevalent types of pesticides employed worldwide [56]. Furthermore, since herbicides are not intended for insect control, manufacturers do not provide warnings regarding the potential impact of these products on bees. Consequently, bees may be exposed to significant levels of herbicides when applied to crops, particularly genetically modified ones during the flowering period [57,58]. Faita et al. (2018) [46] investigated the impact of sublethal doses of the herbicide Roundup^®^ on the hypopharyngeal glands of nursing worker bees and its influence on royal jelly production. The researchers determined that the herbicide induced alterations in the cellular ultrastructure of these glands, leading to premature degeneration of the rough endoplasmic reticulum and significant morphological and structural modifications in the mitochondria. It was emphasized that these discoveries can potentially harm the growth and viability of bee colonies. In a study by Martínez-Domínguez et al. (2016) [59], a multi-class method was developed to identify and quantify more than 260 toxic substances, including pesticides. Eight nutraceutical products (two capsules and six liquid presentations) were analyzed; only one sample was contaminated with propachlor (14.9 μg/kg), but with a concentration below the corresponding MRLs (20.0 μg/kg). The authors emphasized that the presence of this herbicide in royal jelly can be attributed to the fact that the tested sample is a combination of royal jelly and pollen. Therefore, it is plausible that the pesticide could be present in the pollen portion of the sample.

### 2.4. Fungicides

Fungicides are chemical substances employed to eliminate or suppress the growth of fungi, which can cause significant harm to crops and pose risks to the health of humans and domestic animals. The majority of fungicides are toxic to humans and have the potential to induce acute or chronic issues if ingested through food consumption [10]. Although some studies have investigated the presence of fungicides in royal jelly [60], they are generally more frequently found in plants or tea [59], or in combination with other pesticides. Most conducted studies have primarily focused on the effects of insecticides, while fungicides have received limited attention [57]. A combination of miticides, fungicides, herbicides, and insecticides are tested in order to evaluate the impact of colony exposure to a multi-pesticide pollen treatment on the nutritional quality of royal jelly [45]. Milone et al. (2021) [45] detected coumaphos (4.5 µg/kg), 2,4-dimethylphenyl formamide) (4.25–5 µg/kg), thymol (79–301 µg/kg) and other residues at trace levels. Carbendazim, a benzimidazole fungicide commonly used to control the *Sclerotinia sclerotiorum* of rape plants during the flowering period, was sprayed on rape flowers, and its residues (77 μg/kg) were analyzed in royal jelly [61]. Similar tests were also performed studying the migration of tebuconazole between wax and royal jelly [62] and triadimefon from rape flowers to apicultural products [63]. Residues of tebuconazole were found in queen cell cups decreasing its concentration over time. The authors suggested that these concentrations do not pose a lethal risk to queens, but sub-lethal effects should be considered, as azole fungicides have synergistic negative effects on honeybees when combined with insecticides. Additionally, traces of triadimefon were detected (4 μg/kg, MRLs = 100 μg/kg), but at concentrations 10 times lower than the residues found in pollen.

## 3. Applied Methodology to Review the Available Literature

The search was carried out by utilizing Scopus, Science Direct, Web of Science and Google Scholar databases, employing a specific sequence of keywords, including “acaricides”, “fungicides”, “herbicides”, “insecticides”, “pesticides”, “contaminant”, “sample treatment” and “royal jelly”. During the initial screening, duplicates, irrelevant studies or lack of data in studies (e.g., lack of sample preparation methodology) on the frequency were removed. Thus, all abstracts (53 papers published up to July 2023) were analyzed to determine their suitability, and those selected underwent comprehensive scrutiny.

**Figure 1:**
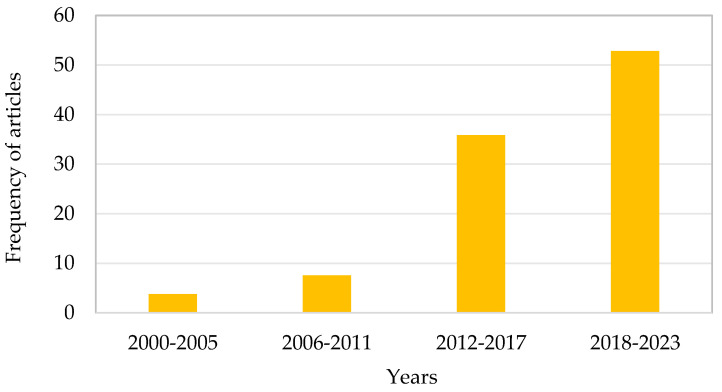
Temporal and frequency evolution of articles focusing on the determination of pesticides in royal jelly. The search has been performed as described in Section 3.

It should be highlighted that databases contain a significant amount of scientific literature related to the analysis of various contaminants in beeswax, honey and pollen [52,64,65,66,67,68]. However, there is limited available information on sample preparation methods for studying pesticides in other matrices, such as propolis or royal jelly [8,13,43]. However, the publication frequency on royal jelly has been increasing, as shown in Figure 1.

**Figure 2:**
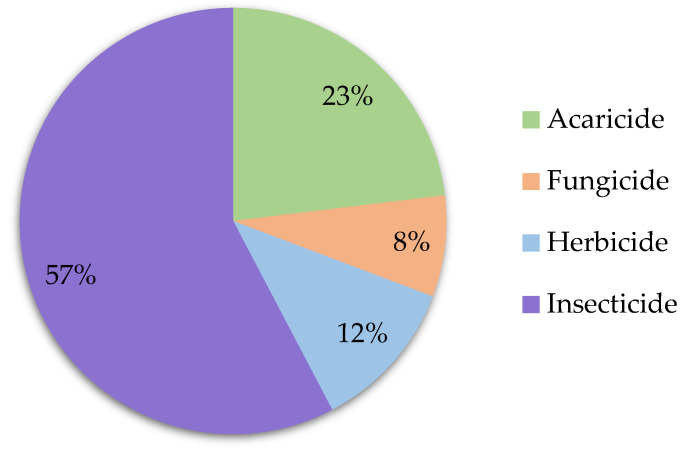
Frequency of class of pesticides analyzed in royal jelly. The search has been performed as described in Section 3.

The initial publications on pesticide residue determination in royal jelly emerged in the year 2000 and have been steadily growing since then. It is worth noting the large number of articles published in the past six years, possibly attributed to the increase in royal jelly production/consumption. Consequently, it is clear that the contamination of royal jelly has not been explored extensively in the literature [15]. This can be attributed to several factors, such as: (i) limited production of royal jelly, (ii) difficulty in collection, (iii) exclusive product, and (iv) lack of knowledge regarding its properties. Given that royal jelly is a high-value food, it is crucial to investigate if it is contaminated in order to ensure safe consumption. It is also important to visualize the frequency of the class of pesticides analyzed in royal jelly (see Figure 2).

**Figure 3:**
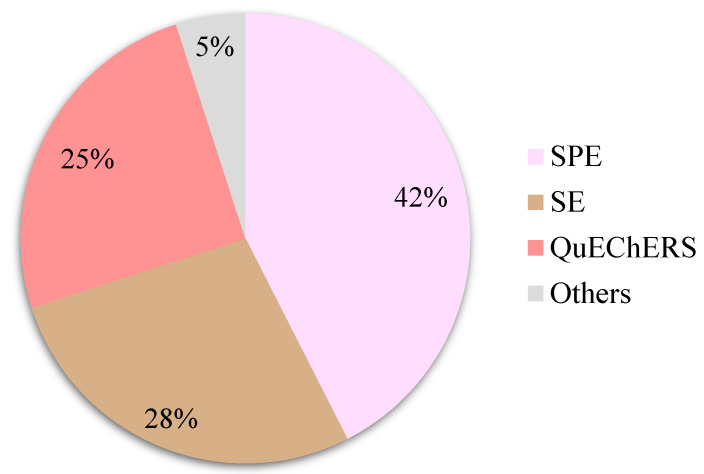
Distribution of sample preparation methods to analyze pesticides in royal jelly. The search has been performed as described in Section 3. QuEChERS: quick, easy, cheap, effective, rugged and safe; SE: solvent extraction; SPE: solid-phase extraction.

Insecticides (57%) are the most extensively studied pesticides in royal jelly, as well as in other bee products. Other secondary contaminants include acaricides (23%), herbicides (12%), and fungicides (8%). Regarding the distribution of sample preparation methods to analyze pesticides in royal jelly (see Figure 3), the publications showed a tendency for SPE (42%), solvent extraction (SE; 28%) and QuEChERS (quick, easy, cheap, effective, rugged and safe; 25%).

SE and SPE have been used in several studies, although they usually either require huge amounts of solvents, or are time-consuming. Therefore, it is not surprising that QuEChERS has attracted the interest of many researchers in the last few years. Finally, it should be mentioned that once the pesticides have been extracted using some of the aforementioned sample preparation methods, the extracts are typically analyzed using chromatographic techniques, either liquid chromatography or gas chromatography, coupled with detectors, primarily electron capture detector [35,69] or mass spectrometry [3,21,47,59,70,71].

## 4. Sample Preparation Methods to Study Pesticides in Royal Jelly

Sample treatment is a crucial step when developing sample preparation methods. Considering bee products and given the diverse matrix components present, it has become imperative to choose different approaches focused on a specific api-product [19]. These methodologies encompass not only the extraction of analytes but also the implementation of effective sample clean-up steps. This ensures the removal of as many matrix components as possible, thus reducing their impact on the evaluation of pesticides. Examining pesticide residues poses a notable difficulty, primarily due to the limited amounts of analytes and the substantial presence of interfering substances that can be co-extracted. Consequently, this frequently leads to errors during experimentation and potential harm to the analytical instruments [18]. Table 2 presents numerous studies that have evaluated sample preparation/extraction methods for different classes of pesticides in royal jelly.

Royal jelly has been described as a highly complex matrix for contaminant extraction [49,72]. Therefore, it is of paramount importance to develop optimized methods with high recovery percentages that minimize matrix effects and, as far as possible, adhere to the principles of green chemistry [54,73,74], particularly, with regard to solvents [75] (see Table 3).

In general, when designing sample treatment methods to extract pesticides in royal jelly, a matrix-matched calibration is typically employed [3,44]. This approach ensures that the calibration standards used for quantification are prepared in a similar matrix as the samples, enhancing the accuracy and reliability of the results. The need to assess the matrix effect prior to quantifying pesticides in royal jelly is often overlooked (see Table 2), but it is crucial for accurately quantifying the samples. Additionally, it is also of utmost importance to consider the physical state of royal jelly, as it can be found in various states [43], which significantly impact the development of sample preparation methods. For example, two methodologies have been developed for analyzing neonicotinoids in products based on royal jelly, one for fresh royal jelly [3], and another for liquid dietary supplements, specifically lyophilized royal jelly in the form of ampoules [19]. Another factor affecting the sample treatment of the royal jelly is its composition, so the extraction solvent and clean-up steps should be optimized to remove sugar and protein interferents that can affect chromatography measurements [47]. Royal jelly’s composition is quite similar to other bee products, and it is subject to variations influenced by factors such as season, geographical regions, honeybee species, and beekeeping methods [33,76]. These differences pose challenges in developing analytical sample treatments and methods, in addition to establishing harmonized MRLs.

Some sample preparation methods for analyzing pesticides in royal jelly, such as the ones described below, have been reported. Despite these, analytical methods for pesticide residue determination in royal jelly are not very well known since it is a bee product of limited production [35]. Therefore, there is a shortage of evidence related to royal jelly, which indicates significant research gaps [8]. To better understand the role of pesticides on queen development and to assess the amount of pesticides to which developing queen bees and humans are likely to be exposed, it is essential to measure potential pesticide levels in royal jelly. Having sample preparation methods and analytical methods that are sufficiently efficient to extract those compounds and clean the extracts for further determination, such as those developed in the literature to up to date (see Table 2), becomes a cornerstone for addressing this issue.

### 4.1. SPE

In the analysis of pesticide residues in royal jelly, SPE has emerged as the predominant technique over the past few decades [10]. The SPE procedure typically yields favorable outcomes in terms of sensitivity, recovery and matrix effect. However, it is important to note that it entails a notable expense in terms of reagents and equipment, particularly due to the cost associated with SPE sorbents. The steps involved in this technique are as follows: (i) sample preparation, (ii) cartridge conditioning, (iii) sample loading, (iv) washing, (v) elution and (vi) evaporation or concentration. Nonetheless, due to the complex composition of royal jelly, which contains multiple substances, direct elution of the cartridges often leads to matrix interference and unclean chromatograms. Consequently, a washing stage is typically necessary to mitigate these issues. Nevertheless, the inclusion of this additional step may potentially result in the loss of certain pesticides, necessitating a careful compromise.

The first mention of utilizing SPE for the analysis of pesticides in royal jelly can be attributed to Karazafiris et al. (2008) [35]. In this study, a novel multi-residue method was developed and validated to analyze four synthetic acaricides commonly used by beekeepers (bromopropylate, coumaphos, malathion and τ-fluvalinate), as well as one pyrethroid, two organochlorine, and two organophosphate insecticides. Sample purification and analyte isolation were achieved by dissolving the matrix in an acetonitrile-water 1:1 *v/v* mixture and passing the supernatant through an octadecylsilane (C_18_) cartridge. The cartridge was previously activated with a mixture of ethyl acetate-hexane (1:1 *v/v*) and acetonitrile followed by water. Ethyl acetate and n-hexane were used for the elution step. The final solution was then analyzed using gas chromatography coupled to a micro-electron capture detector (GC-μECD). An identical protocol using matrix-matched calibration was then used by Martínez-Domínguez et al. (2014) [43] to extract pesticides (*n =* 127; 70–120% recoveries) from royal jelly, comparing it with QuEChERS approaches. A similar protocol was also used by Karazafiris et al. (2022) [39] to assess synthetic acaricide residues (coumaphos and τ-fluvalinate) but they dissolved the sample in an ethanol:water 1:1 *v/v* mixture [77]. Notardonato et al. (2014, 2016) [54,55] conducted two studies where they extracted τ-fluvalinate, fipronil, bromopropylate, amitraz and coumaphos. In one study, they used an acetone: dichloromethane mixture and an adsorbent macroreticular matrix of styrene-divinylbenzene (XAD-2 sorbent), while in the other study, they utilized a toluene solvent and carbograph 1 cartridge, respectively. Since royal jelly is a rather polar matrix with a high-water content, an acetonitrile–water mixture was once again selected in other studies as the appropriate solvent for diluting the sample. The matrix was dissolved using a mixture of acetonitrile and water (1:1, *v*/*v*) and then passed through a C_18_ cartridge [30,78]. Water and methanol were used for cartridge conditioning, while ethyl acetate and hexane were employed for elution. The extraction procedure for amitraz in royal jelly is typically similar to that of τ-fluvalinate. Firstly, it is extracted using an organic solvent (e.g., acetonitrile:water 1:1, *v/v*) after diluting the sample with a buffer due to the amitraz’s sensitivity to acidic conditions. Subsequently, SPE cartridges with different sorbents, such as C_18_, are utilized to clean up the extract. Finally, the extract is concentrated under a nitrogen stream or using a rotary evaporator and then reconstituted for subsequent quantification.

The detection of nitrofurans and derived compounds has also involved the use of SPE approaches. Royal jelly was acidified with hydrochloric acid and trifluoroacetic acid [30]. The pH was then adjusted to 7.5 by adding 1M NaOH, and the supernatant was passed through a polymeric cartridge. For conditioning, 5 mL of methanol and 5 mL of deionized water were used, followed by 10 mL of deionized water for cleaning and 10 mL of ethyl acetate for elution. Similarly, Li et al. (2018) [79] developed a method for the simultaneous determination of seven high-risk pesticides (τ-fluvalinate, triadimenol, coumaphos, haloxyfop, carbendazim, thiophanate-ethyl and thiophanate-methyl) by extracting royal jelly with acetonitrile under alkaline conditions. After dehydration with anhydrous sodium sulfate, the extracts were enriched and purified through polymeric cartridges. Neonicotinoids were analyzed in liquid dietary supplements based on royal jelly using a methodology based on SPE with the utilization of polymeric cartridges [19]. Initially, an attempt was made to extrapolate the QuEChERS method, which had worked well for other bee products (such as honey and pollen), but a difficult-to-remove interface was obtained. Therefore, SPE was tested as an alternative method, as it had been shown to be effective for honey analysis [80]. Valverde et al. (2022) [19] determined that SPE with polymeric cartridges was a satisfactory procedure, achieving recoveries ranging from 85% to 107% using 3.0 g of sample diluted in 10 mL of ammonium formate. The conditioning step involved the use of 5 mL of methanol and 5 mL of water, followed by a 5-minute drying period. Elution was performed using 2 mL of a mixture of methanol and ethyl acetate (70:30, *v/v*). Subsequently, the solution was evaporated to dryness at 60 °C and reconstituted with 1 mL of a mixture of methanol and water (80:20, *v/v*) [3]. The use of polymeric cartridges was similarly used by Li et al. (2017) [61,63] who performed a classic method consisting of three steps. Firstly, they extracted carbendazim and triadimefon from 2.0 g of royal jelly using a water:methanol mixture. Next, they performed clean-up using the dispersive solid-phase extraction technique with an Oasis HLB cartridge. The conditioning involved using 5 mL of methanol and 5 mL of water, followed by 15 mL of water for washing, and finally 10 mL of ethanol for eluting. This SPE approach was also attempted for fresh royal jelly, but it was not suitable for this matrix because the combination with ammonium formate resulted in a viscous solution that caused cartridge clogging [19]. Other authors, such as Hou et al. (2019) [47], have also successfully employed SPE in the study of neonicotinoids, proposing a different three-step sample treatment: (i) dilution with 10 mL of water and precipitation, (ii) extraction with methanol, and (iii) clean-up step based on a SPE with polymeric cartridges. Different SPE sorbents were tested (C_18_ and polymerics), and it was found that the best results in terms of recovery (72–107%) and reproducibility were obtained with a polymeric sorbent. The conditioning involved using 5 mL of water and 5 mL of methanol, 5 mL of methanol: water 1:9 *v/v* for washing, and 5 mL methanol for elution. In addition, in the literature, diatomaceous-based cartridges have been tested for studying insecticides, fungicides and herbicides [42]. First, 1.0 g of royal jelly was mixed with 20 mL of an acetone: water mixture (3:1, *v/v*), and then 5 mL of sodium chloride-solution (20%) was added. The sample was loaded into a diatomaceous earth cartridge and eluted with 10 mL of dichloromethane. Despite achieving nearly 100% recovery in most cases, this methodology required a substantial amount of solvent volume and time.

### 4.2. SE

In this technique, the sample is dissolved in water or water–alcohol mixtures. Once the sample is diluted, it undergoes extraction using appropriate organic solvents. This extraction step aids in isolating the analyte while removing a significant portion of co-extractives. Some variations in this method involve acidifying the sample [81] or utilizing ultrasound [82] to enhance efficiency. However, it is important to consider certain limitations of the SE technique. The process involves the use of substantial amounts of organic solvents, which can have adverse effects on the environment. Moreover, the cost associated with this method is relatively high due to the significant quantity of solvents required. Additionally, analyzing a sample using SE can be time-consuming, and automating the process poses challenges. Despite these aspects, SE has demonstrated satisfactory results in various pesticide determination methods for analyzing royal jelly.

Balayannis et al. (2001) [21] published the first research on residue analysis in royal jelly using SE and gas chromatography–mass spectrometry (GC-MS), which involved a high number of steps, a large number of solvents (207 mL in total) and a substantial sample quantity (5.0 g). The method included sample dilution with a mixture of isopropyl alcohol and acetonitrile, followed by centrifugations for phase separation and extraction steps with methylene chloride. Nitroimidazoles, compounds that have been widely used for the treatment and prophylaxis of certain bacterial and protozoal diseases in animals, were also studied in royal jelly [72]. A total of 2.0 g of royal jelly was alkalinized with a sodium hydroxide solution to dissociate the target analytes from the matrix, followed by two liquid–liquid extractions using 10 mL of ethyl acetate. After evaporation and reconstitution, the extract was injected into the chromatographic system. In contrast to other methods described in the literature, where SPE using cationic, anionic, C_18_ or polymeric sorbents is frequently employed for the analysis of these analytes [83,84], this study proposed a simple method with a reduced number of steps, effectively minimizing matrix effects. Good quantification results were achieved using highly selective reaction monitoring by high performance liquid chromatography–tandem mass spectrometry. Regarding the solvent choice, ethyl acetate, acetonitrile, dichloromethane, or toluene are commonly used for the extraction of nitroimidazole residues in various matrices. However, considering the significant toxicity of dichloromethane and the temperature sensitivity of nitroimidazoles, which can result in the loss of analytes during evaporation, ethyl acetate was selected as the best extraction solvent. Ethyl acetate possesses a relatively low boiling point and low toxicity, making it a suitable choice for the extraction process.

A variation in SE was also tested for the study of insecticides in royal jelly. This method was based on a technique similar to the initial step of the QuEChERS method, assisted by ultrasounds [34]. Specifically, a determination at a trace level of two neonicotinoid insecticide residues (thiamethoxam and clothianidin) in royal jelly was performed using salting-out liquid–liquid extraction, resulting in satisfactory recoveries (94–105%). The salting-out microextraction process involved the addition of 100 μL of water, 1 mL acetonitrile and 300 mg acetate buffer. One of the authors’ objectives was to reduce both the mass of the test sample to 100 mg and the current limit of quantification. A low solvent expenditure was achieved, which limited the method to a single SE. Additionally, in the dispersive phase purification step, primary secondary amine (PSA), C_18_ and the addition of a small volume of hexane were tested to remove some impurities that could cause analytical interferences and inhibit the signal. However, this step was relatively meticulous and repetitive (weighing the dispersive phase as a powder, risk of loss or contamination during handling), so it was discarded and the possibility of adding a freezing step was explored. Thus, a purification step using freezing (15 h) to precipitate interfering compounds (sugar or lipid type). Simpler methodologies employing fewer steps, but higher volumes of organic solvents have also been described [69]. Skerl et al. (2010) [69] used 0.5 g of royal jelly and 90 mL of a n-hexane and isopropanol mixture to extract coumaphos. The upper phase containing hexane was filtered, evaporated and subsequently dissolved.

### 4.3. QuEChERS

QuEChERS, a novel technique for determining pesticide residues in food analysis, was developed and validated by Anastassiades et al. (2003) [85] and quickly adopted by numerous laboratories to study pesticides in bee products [86,87]. The QuEChERS method uses a combination of liquid–liquid extraction, mainly acidified acetonitrile [9], and solid-phase dispersion sorbents (see Table 4) and salts (e.g., magnesium sulphate or sodium acetate) to extract and then clean the extract.

Over the years, the QuEChERS procedure has been modified and used for the determination of pesticide residues in honey [88], wax [89], pollen [86], bumblebees [90] and royal jelly [9,41,44,91,92]. The insecticide pyriproxyfen and its four metabolites were extracted from royal jelly using the QuEChERS method [70]. A total of 5 g was mixed with water, n-hexane (to remove lipid interferences), 1% formic acid in acetonitrile and some salts and sorbents (sodium chloride, magnesium sulphate and PSA). Despite the good recoveries obtained (78–98%), the matrix effect ranged from −37 to −100%, indicating that all signals were suppressed, and a matrix-matched calibration curve should be used. Moreover, the low volume of solvents, the use of a long 24-hour freezing period and the large quantity of sample required further optimization of the sample preparation conditions. Raimets et al. (2022) [62] followed a similar procedure for the extraction of tebuconazole. 2 g of royal jelly were mixed with acetonitrile and water. Then, a mixture of trisodium citrate dihydrate (1 g), sodium chloride (1 g), disodium hydrogen citrate sesquihydrate (0.5 g) and anhydrous magnesium sulphate (4 g) was added to induce analyte transfer, remove any remaining water and stabilize the pH. After a freezing step (−70 °C; 30 min), the extract was transferred into tubes containing anhydrous magnesium sulphate (900 mg), PSA (150 mg) and C_18_ sorbent (150 mg). The utilization of a substantial quantity of salts underscores that the complex composition of royal jelly needs the use of these reagents to effectively cleanse the matrix from interferents, primarily sugars, organic acids and pigments [9]. In other studies, royal jelly was buffered with 0.2 M dibasic sodium phosphate (pH 9) because some pesticides were pH-dependent, existing in neutral or ionized form in different pH media [44]. The feasibility of using Association of Official Analytical Chemists (AOAC) extraction kits and an English QuEChERS reagent kit was also tested.

The success of the extraction procedure relied on efficient degreasing and deproteinization due to the presence of interferences such as proteins, fats, lactose, organic acids, and amino acids in the tested royal jelly. Thus, Zhang et al. (2016) [71] decided to use a mixed solution of 0.1 M citric acid and 0.2 M sodium dihydrogen phosphate to hydrolyze and precipitate proteins in the matrix. Different solvents (acetonitrile, acetic acid in acetonitrile, methanol, ethyl acetate and dichloromethane) and their combinations were tested for analyte extraction. It was found that better recoveries were achieved when acetonitrile acidified with acetic acid was used. In general, certain salts like sodium chlorate, sodium acetate, anhydrous magnesium sulfate and anhydrous sodium sulfate, are utilized as salting-out agents to separate drug residues into the organic layer. The authors opted to use 2 g of sodium chloride and 2 g of sodium sulfate, which resulted in excellent recoveries. Additionally, they employed 200 mg of NH_2_ sorbents for matrix solid-phase dispersion, as it facilitated the simultaneous retention of a wide range of hydrophilic and lipophilic compounds. Martínez-Domínguez et al. (2016) [59] preferred the “dilute and shoot” method over the QuEChERS approach. The acetate QuEChERS method did not yield satisfactory results in terms of compound recoveries for all the targeted 260 molecules while “dilute and shoot” can be used to determine a large number of pesticides simultaneously. In their approach, 7.5 mL of acetonitrile acidified with formic acid (1%, *v/v*) was used for the extraction, followed by clean-up step zirconium dioxide-based sorbent (Z-Sep^+^) to reduce the matrix effect by removing phospholipids and carboxylic acids. Although the analytical workflow was well-suited for comprehensive substance screening in royal jelly, it should be noted that the initial test sample size was relatively large (2.5 g), and the overall sample preparation method was time-consuming (2 h only for shaking). The same conclusions regarding the unfeasibility of using QuEChERS were obtained by Valverde et al. (2018) [3] when they studied neonicotinoids in royal jelly samples. In fact, Martínez-Domínguez et al. (2014) [43] had already emphasized in previous years that the SPE methodology was preferred over the QuEChERS method. A total of 2 g of royal jelly was weighed, and 8 g of water was added to dissolve it. Then, 10 mL of a mixture of acetonitrile and acetic acid at 1% (*v/v*) and a salt mixture (4 g of magnesium sulfate and 1 g of sodium acetate for the American version and 4 g of magnesium sulphate, 1 g of sodium chloride, 1 g of sodium citrate dihydrate and 0.5 g of disodium hydrogen citrate sesquihydrate for the European version) were added. The effect of adding additional clean-up steps (PSA, graphitized carbon black (GCB) and Florisil) was evaluated, but recoveries were not acceptable for all concentration levels studied, so the QuEChERS sample preparation method was discarded.

### 4.4. Other Sample Preparation Methods

Additionally, other methodologies have been employed to isolate pesticides from royal jelly. For example, Tananaki et al. (2009) [50] employed a purge and trap system to isolate *p*-DCB from royal jelly. In this method, the compound molecules were extracted from the aqueous royal jelly solution by purging with helium gas at a flow rate of 40 mL/min for 40 min, while maintaining the sample temperature at 40 °C. The extracted molecules were then absorbed on Tenax resin. The dispersive liquid–liquid microextraction (DLLME) technique may also be a feasible alternative for studying pesticides in royal jelly, although it has only been used in a single study thus far [3]. The process involves several steps. First, the sample containing pesticides is prepared through pre-treatments such as homogenization, filtration or dilution as required. Then, an extracting solvent is added to the sample, typically a mixture of an organic and a dispersive solvent. This mixture aids in dispersing the organic solvent into microdroplets. The solution is then vigorously agitated to ensure dispersion and facilitate the transfer of pesticides from the sample to the organic solvent. After that, centrifugation is performed to separate the phases, the extraction phase containing the target analytes is collected, evaporated or concentrated for subsequent reconstitution. This DLLME procedure has proven successful in studying neonicotinoid insecticides in honey liquors [93]. As a result, researchers have extended the use of this sample preparation method to other matrices, such as fresh royal jelly [3]. Valverde et al. (2018) [3] conducted tests using various solvents, such as acetonitrile, methanol and ethanol, for extraction purposes, as it is a crucial parameter in optimizing the DLLME process. Throughout their study, they discovered that the combination of chloroform as the extraction solvent and acetonitrile as the dispersive solvent, along with two extraction steps, resulted in the highest extraction efficiency. After the second extraction, both chloroform extracts were combined, evaporated and reconstituted using a mixture of methanol and water (80:20, *v/v*). All the neonicotinoids studied exhibited acceptable recovery values (85–107%) and quantification limits (3.7–9.4 μg/kg). These limits were found to be significantly lower than the MRLs established by the European Commission, without any notable impact on the matrix effect for certain analytes [11,19].

## 5. Future Perspectives

According to the comprehensive review of the literature, extensive research has been conducted over the past decades on api-products, primarily focusing on their nutritional value and scientifically proven therapeutic benefits. Numerous published reports have also addressed quality control, safety parameters and the validation of methodologies, potentially paving the way for bee products to establish a significant global market presence. However, there has been a notable absence of discussion regarding different sample preparation methods for studying pesticides associated with royal jelly.

Despite being recognized as a prominent superfood due to its remarkable functionality and nutraceutical properties, our present study revealed that royal jelly may contain various pesticides. To mitigate or eliminate potential contaminants in royal jelly, we suggest implementing several measures. Firstly, it is crucial to carefully restrict the foraging area of honeybees to ensure the harvest of safer and higher quality api-products. Secondly, it is essential to develop strict guidelines concerning pesticide usage and enforce compliance with good agricultural practices. This includes regular monitoring and adherence to safe pesticide application methods, as well as considering alternative, more environmentally friendly pest control approaches. Lastly, adhering to good hygienic sanitary practices during beekeeping is important to prevent the transmission of pathogens and veterinary drug residues into bee products.

Furthermore, despite the increasing amount of technical and scientifically driven research in this domain, there still exists a substantial lack of information concerning royal jelly and other api-products. Even after reviewing all the studies, the current information remains insufficient. Consequently, this work aims to inspire researchers to conduct further studies in this area, thereby addressing the existing knowledge gaps and advancing our understanding of the contamination of royal jelly.

Moreover, methods for sample preparation should be specifically designed and validated for royal jelly, rather than using general and multi-residue methods applied to pesticides in other beehive products. This approach might help explain the variability of results between positive and negative samples found in royal jelly. By tailoring the sample preparation techniques to the unique characteristics of royal jelly, we can improve the accuracy and reliability of pesticide detection, ensuring more consistent and meaningful outcomes in our analyses.

## 6. Conclusions

The exposure of bees to pesticides during pollination or beekeeping practices poses a danger to both the environment and consumers. While the majority of studies have primarily focused on analyzing pesticide residues in bee pollen, beeswax and honey, it is pivotal to note that these residues not only persist in these matrices but can also be transferred to other components of the beehive, including royal jelly. Monitoring pesticide residues in bee products, particularly royal jelly, is key not only to ensure the safety of these products for consumers, including honeybees and humans, but also to protect the environment. It is a well-known fact that the daily consumption of royal jelly by a person is minimal, and even if there is a chance of pesticide residue contamination, the risk to humans is low. However, when it comes to royal jelly contamination by pesticides migrating from wax combs, it can harm the rearing of the bee brood and the survival of the entire colony. Therefore, it is necessary to investigate the transfer of pesticides from wax to larval food and assess its impact on the colony’s well-being and long-term survival. In order to detect them in royal jelly, various sample preparation methods were proposed in the literature such as SPE, SE and QuEChERS. The data and information compiled in this review aimed to facilitate the extraction and determination of pesticides in royal jelly, providing valuable insights for researchers. However, the available literature on this topic is limited, and greater efforts should be made to monitor pesticide residues in royal jelly, considering the significant implications associated with this issue.

## Figures and Tables

**Table 1:** Classification of the most common pesticides detected in royal jelly according to their mode of action.

Family of Pesticide	Target Pests/Function	Pesticide Example
Acaricides	Kill mites that feed on plants and animals	Amitraz
Fungicides	Kill fungi (including blights, mildews, molds and rusts)	Tebuconazole
Herbicides	Kill weeds and other plants that grow where they are not wanted	Propachlor
Insecticides	Kill insects and other arthropods	Dinotefuran

**Table 2:** Representative works concerning pesticides and sample preparation methods to study them in royal jelly-based products.

Pesticide Class	N	Extraction Method	Reagents^TOS^	OST	VM	%R	ME	LOQ (μg/kg)	Residues (μg/kg)	Analytical Technique	Reference
A, H, F, I	127	SPE	0.5 g, 10 mL ACN:H_2_O, 5 mL EtOAc:HX, 3 mL ACN, 2 mL EtOAc, 2 mL HX	Yes	Yes	70–120	Yes	<10	Not found	GC-MS/MS	[43]
A, I	8	SPE	0.5 g, 10 mL ACN:H_2_O, 4 mL EtOAc:HX, 3 mL ACN, 2 mL EtOAc, 2 mL HX	No	Yes	80–91	Yes	3–5	NS	GC-μECD	[35]
I	10	SPE	2 g, 20 mL MeOH	Yes	Yes	73–107	Yes	0.25–5	Not found	HPLC-MS/MS	[47]
I, F, H	13	SPE	1 g, 20 mL AC: H_2_O, 100 mL DCM	No	Yes	74–147	Yes	0.5–5	34–920	HPLC-MS/MS, GC-MS/MS	[42]
A	5	SPE	100 mL AC:DCM	Yes	Yes	81–102	Yes	8–48	81	GC-MS	[54]
A	5	SPE	Toluene	Yes	Yes	99–106	Yes	4.6–9.4	NS	GC-MS	[55]
I	7	SPE, DLLME	3 g, 5 mL MeOH, 2 mL MeOH:EtOAc 0.1 g, 1 mL ACN, 0.25 mL CH	Yes	Yes	83–109	Yes	2.5–9.5	Not found	HPLC-MS/MS	[3]
I	2	SE	0.1 g, 1 mL ACN	Yes	Yes	95–104	Yes	0.25	0.15–0.25	HPLC-MS/MS	[34]
F	2	SE	2 g, 40 mL MeOH	Yes	Yes	100–116	Yes	10	4–77	HPLC-MS/MS	[61]
A	2	SE	5 g, 20 mL ^i^PrOH, 95 mL ACN, 30 mL HCl, 90 mL MC	No	No	82–94	NS	NS	10–92	GC-NPD	[21]
A	1	SE	0.5 g, 90 mL HX: ^i^PrOH	No	No	80–98	NS	NS	170–400	GC-ECD	[69]
H, F	90	QuEChERS	1 g, 20 mL of 5% AA in ACN	Yes	Yes	70–120	Yes	0.21–20	<20	HPLC-MS	[71]
I	1	QuEChERS	5 g, 3 mL HX, 5 mL FA in ACN	Yes	Yes	74–115	Yes	1	NS	HPLC-MS/MS	[70]
F	1	QuEChERS	2 g, 10 mL ACN	No	No	NS	NS	NS	80	HPLC-MS/MS	[62]
A	5	QuEChERS	2 g, 12 mL ACN	Yes	Yes	68–106	Yes	1–5	3.6–3.9	HPLC-MS/MS	[44]

^TOS^: Total Organic Solvents; A: acaricide; AA: acetic acid; AC: acetone; ACN: acetonitrile; CH: chloroform; DCM: dichloromethane; DLLME: dispersive liquid–liquid microextraction; EtOAc: ethyl acetate; F: fungicide; FA: formic acid; GC-µECD: gas chromatography coupled with micro electron capture detector; GC-ECD: gas chromatography coupled with electron capture detector; GC-MS/MS: gas chromatography coupled with tandem mass spectrometry; GC-MS: gas chromatography coupled with mass spectrometry; GC-NPD: gas chromatography nitrogen-phosphorus detector; H: herbicide; HCl: hydrochloric acid; HPLC-MS: high performance liquid chromatography coupled with mass spectrometry; HPLC-MS/MS: high performance liquid chromatography coupled with tandem mass spectrometry; HX: hexane; LOQ: limit of quantification; I: insecticide; MC: methylene chloride; ME: matrix effect; MeOH: methanol; N: number of analytes studied; NS: not specified; OST: optimization of sample treatment; ^i^PrOH: iso-propanol; QuEChERS: quick, easy, cheap, effective, rugged and safe; %R: percentage of recoveries; SE: solvent extraction; SPE: solid phase extraction; VM: validation of the method.

**Table 3:** Solvents most frequently used for the dilution/extraction of pesticides from royal jelly.

Solvents	Safety Score *
Acetonitrile	P
Ethanol	R
Acetone	R
Water	R
Isopropyl alcohol	R
Acetic acid	P
Formic acid	P
Hexane	H
Dichloromethane	H
Toluene	P
Ethyl acetate	R
Methanol	R

* Classification performed according to CHEM21 solvent guide [75]. R: recommended; P: problematic; H: hazardous.

**Table 4:** Typical clean-up sorbents for analysis of pesticides in royal jelly samples. Adapted from Hrynko et al. [87].

Sorbent	Application
Florisil	- Polar - Isolation of hydrophilic, polar substances from non-polar mixtures - Removes lipids, waxes, oils
Octadecylsilane (C_18_)	- Lipophilic character - Binds fats through hydrophobic interactions - Removes non-polar interferences such as lipids or sterols
Primary Secondary Amine (PSA)	- It is a silica modification - Removes sugars, organic acids, fatty acids and certain pigments
Z-Sep	- Is the commercial name of a mixture of two sorbents, C_18_ and silica coated with zirconium dioxide, the proportion of ZrO_2_/C_18_ is 2/5 - Acts as a Lewis acid, attracting compounds with electron donating groups - Removes fats
Graphitized Carbon Black (GCB)	- Known as graphitized soot - Finds application in removal of polyphenols, pigments and dyes - Shows strong affinity to planar molecules–multiple bond systems and aromatic structures - GCBs are made of a nearly homogeneous surface of graphite-like carbon atoms with a surface of oxygen complexes

## Data Availability

The data that support this study are available from the corresponding author upon request.

## Abbreviations

AOAC—Association of Official Analytical Chemists; C_18_—Octadecylsilane; DLLME—Dispersive Liquid-Liquid Microextraction; EU—European Union; FAO—Food and Agriculture Organization; MRL—Maximum Residue Limit; GCB—Graphitized Carbon Black; GC-MS—Gas Chromatography Mass Spectrometry; GC-μECD—Gas Chromatography coupled to Micro-electron Capture Detector; p-DCB—*p*-dichlorobenzene; PSA—Primary Secondary Amine; QuEChERS—Quick, Easy, Cheap, Effective, Rugged and Safe; SE—Solvent Extraction; SPE—Solid-Phase Extraction.
